# Broad-Spectrum Antiviral Activity of an Ankyrin Repeat Protein on Viral Assembly against Chimeric NL4-3 Viruses Carrying Gag/PR Derived from Circulating Strains among Northern Thai Patients

**DOI:** 10.3390/v10110625

**Published:** 2018-11-13

**Authors:** Supachai Sakkhachornphop, Sudarat Hadpech, Tanchanok Wisitponchai, Chansunee Panto, Doungnapa Kantamala, Utaiwan Utaipat, Jutarat Praparattanapan, Wilai Kotarathitithum, Sineenart Taejaroenkul, Umpa Yasamut, Koollawat Chupradit, Sutpirat Moonmuang, Vannajan Sanghiran Lee, Khuanchai Suparatpinyo, Chatchai Tayapiwatana

**Affiliations:** 1 Research Institute for Health Sciences, Chiang Mai University, Chiang Mai 50200, Thailand; ssakkhachornphop@yahoo.com (S.S.); chansunee_panto@yahoo.com (C.P.); doungnapak@hotmail.com (D.K.); utaipatu@gmail.com (U.U.); sineenart.t@cmu.ac.th (S.T.); khuanchai.s@gmail.com (K.S.); 2 Center of Biomolecular Therapy and Diagnostic, Faculty of Associated Medical Sciences, Chiang Mai University, Chiang Mai 50200, Thailand; winnypuf@live.com (S.H.); miisuii@hotmail.com (T.W.); umpa_119@hotmail.com (U.Y.); kool_krub@msn.com (K.C.); sutpirat_mo@hotmail.com (S.M.); 3 Faculty of Pharmaceutical Sciences, Burapha University, Chonburi 20131, Thailand; 4 Department of Medicine, Faculty of Medicine, Chiang Mai University, Chiang Mai 50200, Thailand; jutarat.praparattanapan@gmail.com (J.P.); wilai.k@cmu.ac.th (W.K.); 5 Division of Clinical Immunology, Department of Medical Technology, Faculty of Associated Medical Sciences, Chiang Mai University, Chiang Mai 50200, Thailand; 6 Department of Chemistry, Faculty of Science, University of Malaya, Kuala Lumpur 50603, Malaysia; vannajan@um.edu.my; 7 Biomedical Technology Research Center, National Center for Genetic Engineering and Biotechnology, National Science and Technology Development Agency, Faculty of Associated Medical Sciences, Chiang Mai University, Chiang Mai 50200, Thailand

**Keywords:** HIV-1, Gag polyprotein, virus assembly inhibitor, ankyrin, protein therapy

## Abstract

Certain proteins have demonstrated proficient human immunodeficiency virus (HIV-1) life cycle disturbance. Recently, the ankyrin repeat protein targeting the HIV-1 capsid, Ank^GAG^1D4, showed a negative effect on the viral assembly of the HIV-1_NL4-3_ laboratory strain. To extend its potential for future clinical application, the activity of Ank^GAG^1D4 in the inhibition of other HIV-1 circulating strains was evaluated. Chimeric NL4-3 viruses carrying patient-derived Gag/PR-coding regions were generated from 131 antiretroviral drug-naïve HIV-1 infected individuals in northern Thailand during 2001–2012. SupT1, a stable T-cell line expressing Ank^GAG^1D4 and ankyrin non-binding control (Ank^A3^2D3), were challenged with these chimeric viruses. The p24CA sequences were analysed and classified using the K-means clustering method. Among all the classes of virus classified using the p24CA sequences, SupT1/Ank^GAG^1D4 demonstrated significantly lower levels of p24CA than SupT1/Ank^A3^2D3, which was found to correlate with the syncytia formation. This result suggests that Ank^GAG^1D4 can significantly interfere with the chimeric viruses derived from patients with different sequences of the p24CA domain. It supports the possibility of ankyrin-based therapy as a broad alternative therapeutic molecule for HIV-1 gene therapy in the future.

## 1. Introduction

Despite the success of human immunodeficiency virus (HIV-1) treatment using highly active antiretroviral therapy (HAART), the complete elimination of the HIV-1 virus from the patient’s body is still not possible. HAART provides effective treatment options for both treatment-naïve and treatment-experienced patients. Nonetheless, it is a lifelong therapy; however, HIV-1 drug-resistant strains have been reported because of a lack of adherence to the therapy. In addition, the toxicity or side effects of antiretroviral drugs result in a decreased quality of life for infected people [1,2,3,4]. Therefore, for these reasons, alternative approaches for HIV-1 therapy are required. Protein therapy is a promising technique that has become an integral and significant part of current medical treatment. Good examples of this next-generation medicine are antibodies and their engineered versions, such as bispecific or multispecific antibodies [5,6,7,8]. In this past decade, during which in vitro selection technology was developed, the selection of a binding protein without immunisation became possible. This led to many non-immunoglobulin scaffold proteins being characterised, used, and engineered to obtain a specific binding candidate [8,9,10].

Recently, our team published the proof of concept of using an ankyrin repeat protein as an antiviral agent [11,12]. This repeat scaffold protein specifically interacts with the p24 capsid domain (p24CA) of HIV-1 Gag polyprotein (Pr55Gag), the so-called Ank^GAG^1D4 domain. Ank^GAG^1D4 was isolated using the phage display technique and consequently designed for the intracellular expression when the gene encoding Ank^GAG^1D4 was placed C-terminal to a myristic signal sequence for membrane targeting. The results showed that this scaffold molecule mediated its antiviral effect at the late stage of the HIV-1 life cycle by interfering with viral assembly [11,12]. The ankyrin repeat protein is one of various protein scaffolds that have been proposed as alternative development platforms to monoclonal antibodies because of the characteristics of this protein family [13]. The advantages of scaffold molecules are such that they transcend the limitations of antibodies, which consequently make them promising candidates for clinical applications. For instance: (i) they are free of disulphide bonds, which allows them to be expressed in the reducing environment of the cytoplasm; (ii) they have high solubility and high thermal stability; (iii) they are smaller than antibody molecules; (iv) they have better tissue penetration and accumulation capacity; and (v) they incur low production or synthesis costs [9,14,15,16]. Interestingly, a designed ankyrin repeat protein, Abicipar pegol, which is specific to vascular endothelial growth factor-A, was approved by the Food and Drug Administration (FDA). A clinical study in patients with wet age-related macular degeneration demonstrated higher binding affinity in the single-digit picomolar level and longer intravitreal half-life in comparison with ranibizumab [17]. Currently, Abicipar pegol is being evaluated in a phase III clinical trial for ophthalmic treatment [17,18,19].

Even though Ank^GAG^1D4 demonstrated very promising negative interference against HIV-1 production, it has been tested only against the HIV-1_NL4-3_ laboratory strain, simian immunodeficiency virus (SIVmac), and simian/human immunodeficiency virus (SHIV) [11,12]. Indeed, HIV-1 is classified into at least nine subtypes (A, B, C, D, F, G, H, J, and K), 58 circulating recombinant forms (CRFs), and various unique recombinant forms (URFs) [20]. In Thailand, CRF01_AE is predominant, followed by subtype B. In addition, reports also suggest that the intermixing of these two subtypes makes them increase in number, for example, CRF15_01B and CRF34_01B [21,22,23,24]. A structural analysis of Ank^GAG^1D4 showed that the binding pockets of Ank^GAG^1D4 specifically bound to helices 1 and 7 of the viral target; and the N-terminal domain of p24CA (NTD^p24CA^) was found to be involved in the Ank^GAG^1D4-NTD^p24CA^ interaction [25]. The target of ankyrin is highly conserved among the viral strains; therefore, in the present study, we explored the broad antiviral function of Ank^GAG^1D4 against the circulating strains in treatment-naïve patients in northern Thailand.

## 2. Materials and Methods

### 2.1. Cells

Human embryonic kidney 293T (HEK293T) cells and SupT1 cells (human T cells, lymphoblastic lymphoma) were obtained from the American Type Culture Collection (ATCC, Manassas, VA, USA). The HEK293T cells were maintained in Dulbecco’s modified Eagle’s medium (DMEM) (Gibco BRL, Grand Island, NY, USA), and the SupT1 cells and Jurkat green fluorescent protein (GFP)-reporter T cells were grown in Roswell Park Memorial Institute (RPMI)-1640 medium (Gibco) supplemented with 100 U/mL of penicillin (Gibco), 100 µg/mL of streptomycin (Gibco), 2 mM of l-glutamine (Gibco), and 10% foetal bovine serum (FBS) (Gibco). All of the cell lines were maintained in a humidified atmosphere at 37 °C in an incubator containing 5% CO_2_.

### 2.2. Viral RNA Extraction and Preparation of the Insert DNA Fragment Encoding the Gag/PR Protein

The viral RNA was extracted from plasma samples using a QIAamp Viral RNA Mini Kit (QIAGEN, Hilden, Germany) according to the manufacturer’s protocol. The aliquots of RNA were kept frozen at −70 °C until use. The gene encoding the Gag/PR protein from the patients was obtained by reverse transcription-PCR (RT-PCR), as previously described [26], starting from HXB2 at nt. 623 to HXB2 at nt. 2849, using the viral RNA template isolated from the plasma samples of 131 antiretroviral drug-naïve HIV-1 infected individuals in northern Thailand during 2001–2012. The DNA products from RT-PCR were then used as templates for second-round PCR in a reaction containing a pair of 100-mer long specific oligonucleotide primers complementary to the sequences of pNL4-3, as previously described [26]. Thereafter, a QIAquick PCR purification kit (QIAGEN) was used to purify the PCR products.

### 2.3. Preparation of Viral Vector Backbone for Chimeric HIV-1 Construction

To construct the chimeric virus, vector pNL4-3∆*gag/PR* was used as the backbone vector. This vector was constructed by the insertion of the specific restriction enzyme *BstE*II at the 5′ end of the *gag* gene, and at 45 bases downstream from the 3′ end of the *PR* gene using the QuikChange II Site-Directed Mutagenesis Kit site (Stratagene, La Jolla, CA, USA). The backbone vector was treated with *BstE*II (Thermo Scientific, Rockford, IL, USA) to remove the wild-type *gag/PR* and allow the vector to self-ligate. Then, the stock backbone vector was prepared by transformation into Top 10 *Escherichia coli* competent cells. The bacterial cells were spread on Luria–Bertani (LB) agar containing 100 µg/mL of ampicillin (Gibco). A Hi-Speed Plasmid Maxi Kit (Qiagen) was used to extract and purify the backbone vector [26].

### 2.4. Production and Titration of Chimeric HIV-1

The amplicons from the second-round PCR and linearised pNL4-3∆*gag/PR* treated with *BstE*II were transfected into the Jurkat GFP-reporter T-cell line (a T-cell line that expresses GFP when the Tat protein from the virus is presented) by electroporation. The cells were further cultured in a T25 flask for 15–30 days to allow the homologous recombination between the vector and the *gag/PR* to be initiated, resulting in the production of chimeric virus from the completed virus. The culture supernatant containing the chimeric virus particles was harvested when the percentage of GFP-positive cells was observed to be about 15–20%. The aliquots of the viruses were kept at −70 °C, and termed the virus stock. The titre of the chimeric virus was then investigated by transduction of the Jurkat GFP-reporter T-cell line using each individual virus stock, followed by monitoring GFP-positive cells at 48 h post-transduction, which would represent the amount of infectious virus particles in the stock.

### 2.5. Production of Pseudotyped Lentiviral Vector to Transfer Ank^GAG^1D4 or Ank^A3^2D3 Coding Genes into SupT1 Cells

VSV-G-pseudotyped lentiviral vector particles harbouring the gene encoding Ank^GAG^1D4, which was designed to fuse with the enhanced green fluorescent protein (EGFP) reporter gene at the C-terminus, were produced in the HEK293T cells using four separate plasmids. Transfection was performed using the calcium phosphate co-transfection method. In brief, HEK293T cells at 70–80% confluence in a 10-cm dish were co-transfected with 10 µg/dish of CGW_Ank^GAG^1D4-EGFP or the CGW_Ank^A3^2D3-EGFP transfer vector, together with the packaging constructs at 6.5 µg/dish of pMDLg/pRRE, 2.5 µg/dish of pRSV-Rev, and 3.5 µg/dish of pMD.2G. At 16 h post-transfection, fresh C-DMEM containing 5% FBS was replaced, and the cells were further incubated. The particles of the lentiviral vector were harvested from the culture supernatant at 24 h and 48 h post-transfection. They were filtered through sterile syringe filters with a 0.45-µm pore size in a Millex-HA filter unit (Merck Millipore, Hessen, Germany). The high titre of the viral vector stocks was prepared by 20% sucrose cushion ultracentrifugation at 100,000× *g*, at 4 °C, for 2 h and 20 min. The viral vector pellets were resuspended in sterile 0.1% bovine serum albumin (BSA) phosphate-buffered saline (PBS), split into 50-µL aliquots, and kept at −70 °C. The viral titre was determined based on the infection of the HEK293T cells with serial dilution of the samples, and the percentage of the EGFP-positive cells at 48 h post-transduction was monitored using flow cytometry (FACSCalibur; BD Biosciences, Le Pont de Claix, France), in which EGFP represented the amount of infectious lentivirus particles [11].

### 2.6. Production of SupT1 Cells Stably Expressing Ank^GAG^1D4 or Ank^A3^2D3

Aliquots of SupT1 cells were transduced with the VSV-G-pseudotyped CGW_Ank^GAG^1D4-EGFP or CGW_Ank^A3^2D3-EGFP vectors at 10 multiplicity of infection (MOI) by spinoculation (centrifugal inoculation) at 2500× *g*, 32 °C for 3 h in a growth medium containing 8 µg/mL of polybrene (Sigma-Aldrich, St. Louis, MO, USA). At 16 h post-transduction, the cells were washed three times with serum-free medium and then maintained at 37 °C and 5% CO_2_ in fresh C-RPMI supplemented with 10% FBS (Gibco), 100 U/mL of penicillin (Gibco), and 100 µg/mL of streptomycin (Gibco). Limiting dilution was performed to obtain the Ank^GAG^1D4 or Ank^A3^2D3-positive cells. Cells were washed three times with PBS, and then resuspended in 1% paraformaldehyde in PBS. The EGFP positive cells were analysed using flow cytometry (FACSCalibur; BD Biosciences).

### 2.7. Inhibitory Effects of Ank^GAG^1D4 Against Gag/PR Chimeric HIV-1

SupT1 cells stably expressing Ank^GAG^1D4 or Ank^A3^2D3 were transduced with 0.2% MOI of the chimeric NL4-3 viruses carrying Gag/PR-coding regions derived from the 131 antiretroviral drug-naïve HIV-1 infected individuals. At 16 h post-infection (hpi), the cells were washed with serum-free medium to remove any excess virus. The cells were further cultured for 13 days. The culture supernatant of day 7 was collected for p24CA enzyme linked immunosorbent assay (ELISA) analysis using Genscreen ULTRA HIV Ag-Ab Assay (Bio-Rad, Redmond, WA, USA).

### 2.8. K-Means Clustering Method

The p24CA levels determined in SupT1/Ank^GAG^1D4 and SupT1/Ank^A3^2D3 transduced with chimeric NL4-3 viruses carrying the Gag/PR-coding regions from the same individual were classified using the K-means clustering method. All 131 chimeric viruses were divided into four clusters by randomly selecting the initial cluster centroid positions. The squared Euclidean distances of all of the individuals to each centroid were measured. Each individual was assigned to the cluster with the closest centroid.

### 2.9. Analysis of Capsid p24CA DNA Sequences

A pair of universal oligonucleotide primers was designed to analyse all of the HIV-1 p24CA DNA sequences. The nucleotide sequences of the forward and the reverse primers are FWD_RIHES_P24; 5′-ggatagaggtaaaagacaccaaggaagc-3′, and REV_RIHES_P24; 5′-ctcattgcctcagccaaaacccttgc-3′, respectively. The DNA fragment encoding the Gag-PR sequence from 131 individual patients was used for p24CA DNA sequencing. The nucleotide sequences of the p24CA DNA from each individual virus were aligned in the VectorNTI software, followed by manual alignment editing using BioEdit. Consequently, all of the p24CA nucleotide sequences were aligned against HXB2, which is a reference viral strain at the NTD^p24CA^ position. The multiple alignment parameters were set using 15 as the gap opening penalty, and 6.66 as the gap extension penalty, and with 40% identity for alignment delay.

Consensus amino acid analysis was performed using BioEdit. The gaps were treated as residues, and were included in the consensus. The maximum threshold frequency for inclusion in the consensus was used for a particular amino acid. To analyse the distribution of the amino acids, a polymorphism was defined as an amino acid that differed from the corresponding consensus amino acid and with a prevalence ≥5%. An amino acid with no polymorphism was considered as a highly conserved amino acid. At the same time, an amino acid with a polymorphism and corresponding consensus amino acid present at <50% was identified as a variable amino acid. Ank^GAG^1D4 binding pockets were defined by protein positions within a minimum Euclidean distance of ≤5Å. Sequence identity was calculated by dividing the number of NTD^p24CA^ identical amino acids by 146 (the number of amino acids in NTD^p24CA^ region).

### 2.10. Statistical Analysis

The statistical analyses for the p24CA level were performed using the paired *t*-test. For the 131 individuals, the paired Student *t*-test was used, whereas the Wilcoxon matched-pairs signed-rank test was applied for the clusters of the p24CA level. Values were considered statistically significant at *p* values of <0.01 (**).

### 2.11. Study Subjects

The study subjects included 131 plasma samples taken from HIV-1 infected patients before receiving antiretroviral drugs from Maharaj Nakorn Chiang Mai Hospital in 2001–2012. The age of the study population was >18 years. All of the plasma samples were stored frozen at −80 °C until use. All of the samples used in this study received full legal approval by the Human Experimentation Committee Research Institute for Health Sciences (RIHES) Chiang Mai University (No. 57/2016).

## 3. Results

### 3.1. Establishment of SupT1 Cells Stably Expressing Ank^GAG^1D4 and Ank^A3^2D3

**Figure 1:**
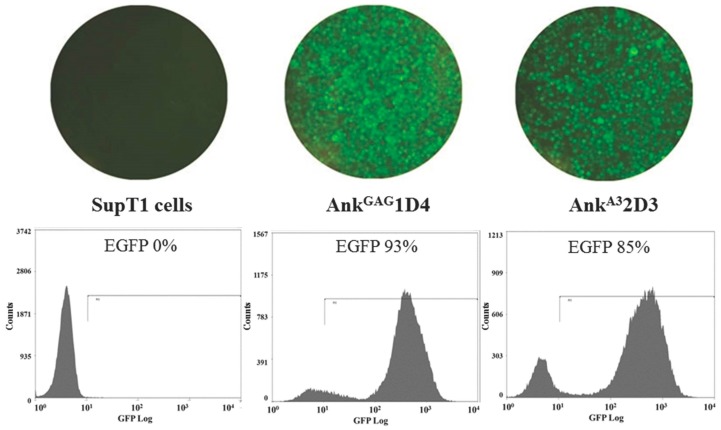
Establishment of SupT1 cells stably expressing Ank^GAG^1D4 and Ank^A3^2D3 fused to an enhanced green fluorescent protein (EGFP) reporter protein. The EGFP reporter protein expression and the morphology of the cells were determined using an inverted fluorescence microscope at 200× magnification (*top panel*) at 48 h post-transduction. The percentages of EGFP-positive cells, which represent the SupT1 cells expressing Ank^GAG^1D4 and Ank^A3^2D3, were investigated using flow cytometry (*bottom panel*).

SupT1 cells were transduced with the VSV-G-pseudotyped CGW_Ank^GAG^1D4-EGFP or CGW_Ank^A3^2D3-EGFP. The EGFP reporter protein expression of the cells was monitored using fluorescence microscopy at 48 h post-transduction. The percentages of the SupT1 cells expressing Ank^GAG^1D4 and Ank^A3^2D3, as analysed using flow cytometry, were 93% and 85%, respectively, as shown in Figure 1.

### 3.2. Effect of Ank^GAG^1D4 Against Gag/PR Chimeric Viruses

**Figure 2:**
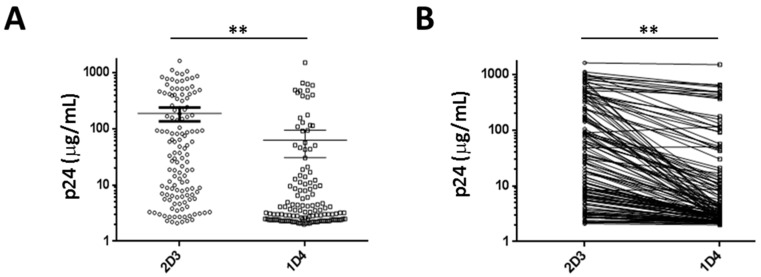
The p24 capsid domain (p24CA) levels in the supernatant collected from SupT1 cells harbouring Ank^GAG^1D4 and Ank^A3^2D3 after infection with 131 Gag/PR chimeric viruses. (**A**) Distribution of p24CA showed that the mean ± SEM of SupT1/Ank^A3^2D3 and SupT1/Ank^GAG^1D4 was 186.5 ± 25.8 µg/mL and 62.2 ± 16.0 µg/mL, respectively. (**B**) Significant reduction of p24CA in SupT1/Ank^GAG^1D4 compared with SupT1/Ank^A3^2D3 by individual samples. ** *p* < 0.01 was determined using a two-tailed paired Student *t*-test. The percentage of samples that experience reduction was 98.47%.

**Figure 3:**
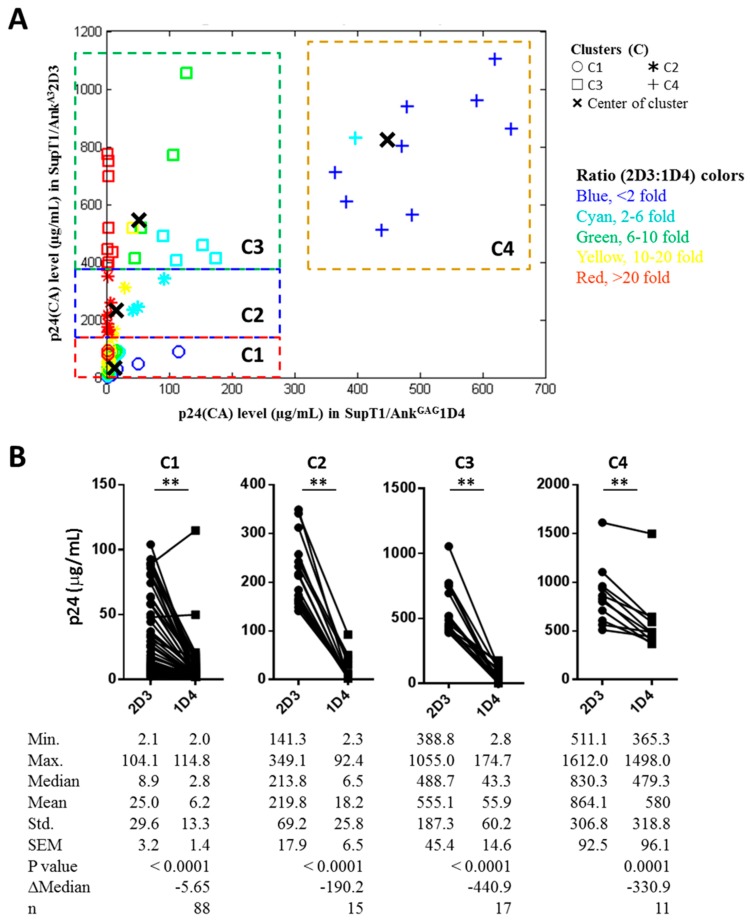
Quantitative analysis of the p24CA levels of the four clusters. (**A**) Four clusters, namely C1, C2, C3, and C4, were classified by the K-means clustering method. In the case of C4, a dataset in which the p24CA levels of SupT1/Ank^GAG^1D4 and SupT1/Ank^A3^2D3 were over 1500 µg/mL was excluded from the plot. (**B**) The p24CA level of the four clusters after being classified by the K-means clustering method. Significant differences (** *p* < 0.01) in the p24CA levels between SupT1/Ank^GAG^1D4 and SupT1/Ank^A3^2D3 were determined using the two-tailed Wilcoxon matched-pairs signed-rank test.

To investigate the antiviral effect of Ank^GAG^1D4 against HIV-1 Gag/PR chimeric viruses, the amount of virus progeny production was determined using the p24CA ELISA assay. The culture supernatant fluids of SupT1/Ank^GAG^1D4 and SupT1/Ank^A3^2D3 (non-binding control) infected with each of the individual Gag/PR chimeric viruses (total = 131 samples) were collected at day 7 post-infection, and assayed for their p24CA levels. The results showed that the mean p24CA level in SupT1/Ank^GAG^1D4 was 62.2 ± 16.0 µg/mL (mean ± SEM, varying from 2.0 µg/mL to 1498.0 µg/mL); meanwhile, the mean p24CA level in SupT1/Ank^A3^2D3 was 186.5 ± 25.8 µg/mL (mean ± SEM, varying from 2.1 µg/mL to 1612.0 µg/mL), as shown in Figure 2A. A single individual sample analysis confirmed that the p24CA level was significantly reduced by the effect of Ank^GAG^1D4 compared with that of Ank^A3^2D3 when challenged with the same Gag/PR chimeric virus (Figure 2B). The wide range of distribution of the p24CA level in the culture supernatant prompted us to classify them into four clusters (C): C1, C2, C3, and C4 (Figure 3A) using the K-means cluster method [27,28]. The cluster plot revealed that low levels of p24CA in SupT1/Ank^GAG^1D4 were found in samples with high and low levels of p24CA in SupT1/Ank^A3^2D3, and that the ratios of p24CA between SupT1/Ank^A3^2D3 and SupT1/Ank^GAG^1D4 were observed to be in the range of <twofold to >20-fold, as shown in Figure 3 (for C1, C2, and C3). Meanwhile, high ratios of p24CA between SupT1/Ank^A3^2D3 and SupT1/Ank^GAG^1D4 were found only in cluster C4 (Figure 3); however, the level of p24CA in SupT1/Ank^GAG^1D4 was lower than that in SupT1/Ank^A3^2D3. The result shows that for all four clusters, the p24CA levels of SupT1/Ank^GAG^1D4 and Ank^GAG^2D3 were significantly different, as shown in Figure 3B. Moreover, the representative samples in each cluster (C1, C2, C3, and C4) were performed to observe the level of intracellular p24CA and viral load harvested from SupT1/Ank^GAG^1D4, and SupT1/Ank^A3^2D3 cells on day 7 post-infection. These results demonstrated that they were consistent with p24 level in culture supernatant described in Supplementary Figures S1 and S2.

**Figure 4:**
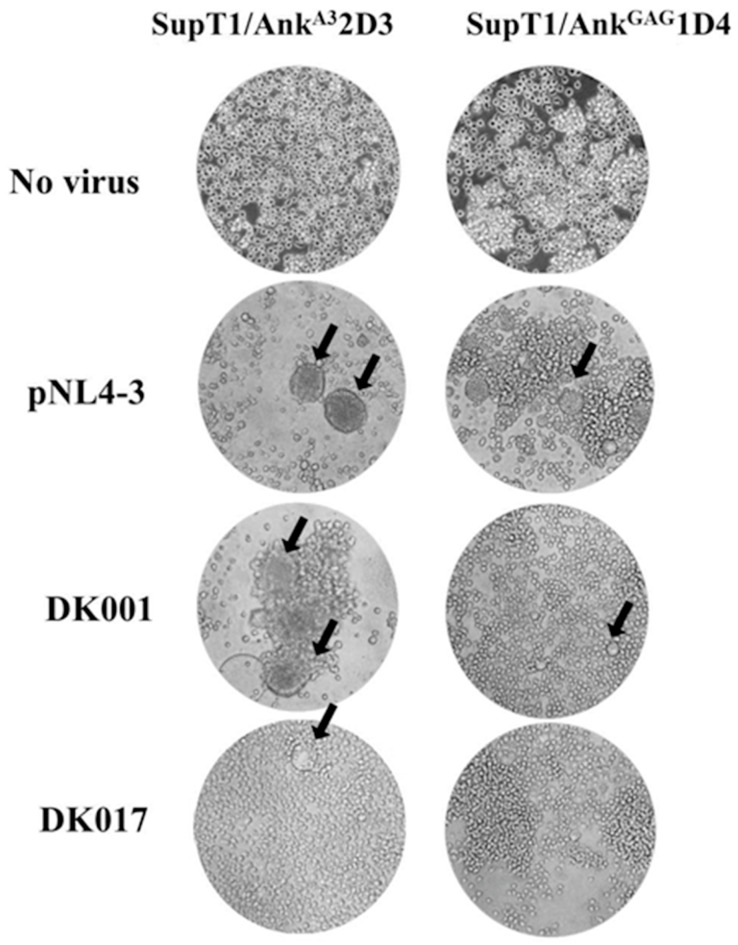
Ank^GAG^1D4 inhibits syncytium formation of SupT1 cells after being infected by Gag/PR chimeric viruses induction. SupT1/Ank^GAG^1D4 and SupT1/AnkG^A3^2D3 were infected with chimeric viruses. The cells were observed at 400× magnification using an inverted microscope. The black arrows point to syncytia. DK001 and DK007 are two representative samples of the Gag/PR chimeric viruses in cluster 1.

In addition, syncytium formation was observed as a sign of infection. As shown in Figure 4 (left panel), the number of syncytia in SupT1/Ank^A3^2D3 was higher than that in SupT1/Ank^GAG^1D4 (right panel). This implied that Ank^GAG^1D4 could inhibit the syncytium formation induced by viral infection. These phenomena were observed in both the cells infected with the HIV-1 NL4-3 laboratory strain and the Gag/PR chimeric viruses (as shown by the two representative samples of the Gag/PR chimeric viruses, DK001 and DK007).

### 3.3. Sequence Identity of NTD^p24CA^

To observe the diversity of NTD^p24CA^, which was the Ank^GAG^1D4 binding target [25], in the 131 Gag/PR chimeric viruses, the sequence identity between individuals was determined. The DNA sequences of the 131 individuals were aligned against the HXB2 positions 1186–1623 and converted to amino acids (p24CA residues 1–146). The sequence identity between individuals varied from 71.0% to 100% (mean ± SD, 96.1 ± 4.3%). From 131 individual chimeras, five identical sequence (IS) patterns of p24CA were shown, IS#1, IS#2, IS#3, IS#4, and IS#5 (Table 1). The sequence identity values between the consensus amino acids and the IS# of 1, 2, 3, 4, 5 were 100% (n = 2), 99.3% (n = 2), 99.3% (n = 8), 97.9% (n = 2), and 97.3% (n = 2), respectively. Whereas the other chimeric viruses inherited the unique sequence of p24CA, they were not included in Table 1. The level of p24CA generated from SupT1/Ank^GAG^1D4 and SupT1/AnkG^A3^2D3 infected with individual viruses from five classified types was informed in Table 1. The levels of p24CA were not associated with the sequence identity in each classified type.

### 3.4. NTD^p24CA^ Amino Acid Distribution

**Figure 5:**
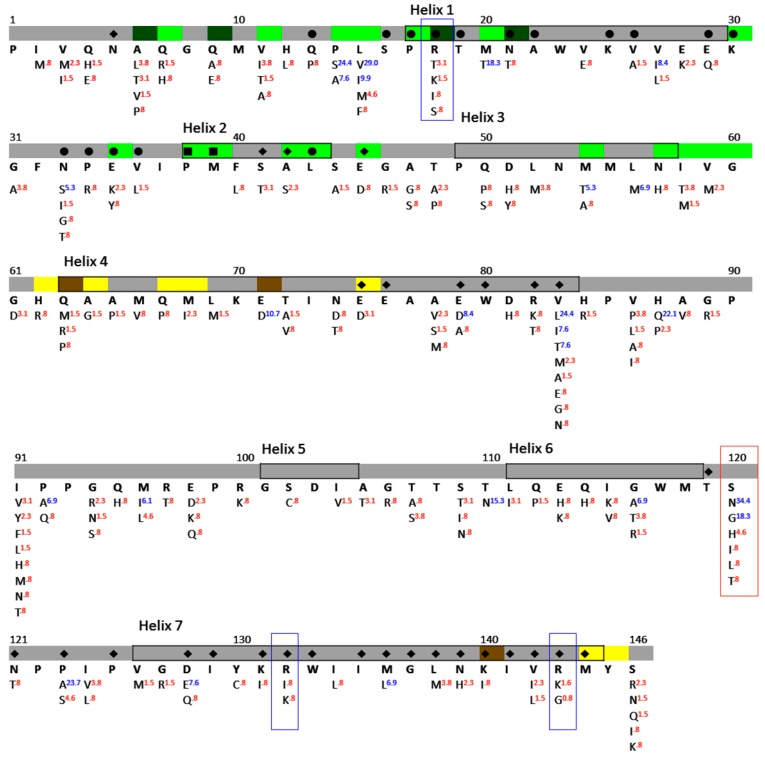
The consensus sequence and amino acid distribution of NTD^p24^ in 131 Gag/PR chimeric viruses. For each NTD^p24^ position, the HXB2 index is shown at the top, followed by the NTD^p24CA^ interface, corresponding consensus amino acid, and prevalence of particular residues. The NTD^p24CA^–NTD^p24CA^ interface and the NTD^p24CA^–CTD^p24CA^ interface (distance < 5 Å) are marked in green and yellow, respectively. The interface residues making the hydrogen bond are in dark green (NTD^p24CA^–NTD^p24CA^) and brown (NTD^p24CA^–CTD^p24CA^). The symbols in colour denote the Ank^GAG^1D4 binding pocket residues. The bullets, diamonds, and boxes are the binding residues in the complexes of Ank^GAG^1D4 bound to helix 1, those bound to helix 7, and those binding both helices, respectively. The corresponding consensus amino acid was defined by the most prevalent of the particular residues. Polymorphisms with proportions ≥5% are indicated using blue superscripts, and red otherwise. The amino acids in the blue box are alternative characters of the key residues interacting with Ank^GAG^1D4, whereas the amino acids in the red box are variable residues.

To reveal the amino acid distribution at the 146 NTD^p24CA^ positions of the 131 individuals, the prevalence of each particular amino acid was observed. A corresponding consensus sequence, which presents the maximum prevalence of the particular amino acid, was built, as shown in Figure 5. Those amino acids that differed from the corresponding consensus amino acid and with prevalence ≥5% were defined as having a polymorphic position. An amino acid position with no polymorphisms present was considered as a highly conserved residue. At the same time, an amino acid position with a polymorphism and prevalence of the consensus amino acid of <50% was defined as being a variable amino acid. It was found that there were 127 (87.0%) highly conserved residues in the 146 NTD^p24CA^ region. Considering that 19 amino acids had polymorphisms, the prevalence of the corresponding consensus amino acid was found to vary from 40.5% to 93.9%, and these percentages could be classified as <50% (residue S120), 50–90% (residues P14, L15, M20, E71, V83, H87, M96, T110, G116, and P123), and >90% (residues V27, N33, M54, L56, E79, P92, D128, and M136). This indicated that most of the NTD^p24CA^ positions among the 131 individuals were highly conserved residues; only one residue (0.7%) was a variable residue (S120).

According to a previous study [25], residues R18, R132, and R143 of NTD^p24CA^ are key amino acids interacting with Ank^GAG^1D4. In Figure 5, the alternative characteristics at positions 18 (R^93.9^, T^3.1^, K^1.5^, I^0.8^, and S^0.8^), 132 (R^98.5^, I^0.8^, and K^0.8^), and 143 (R^97.7^, G^0.8^, and K^1.5^) could be identified as highly conserved residues. It should be noted that the superscripts indicate the residue’s prevalence at that particular position in the alignment. Moreover, 11 out of the 131 individuals had different amino acids from arginine residues. However, simultaneous mutation was not found at these three positions, as shown in Table 2.

**Figure 6:**
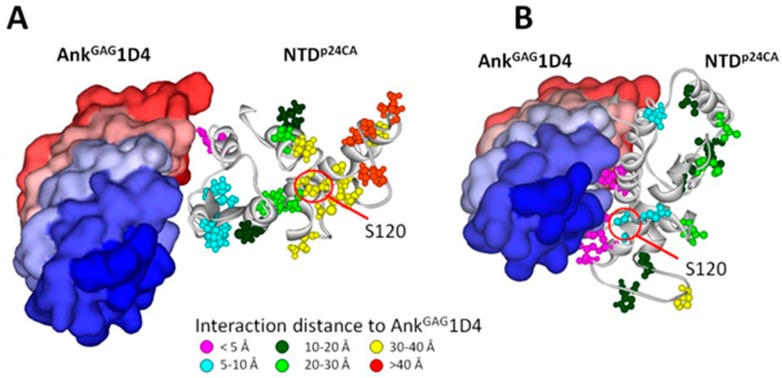
Nineteen amino acids having polymorphic residues present in the docking complexes of Ank^GAG^1D4 (PDB code: 4HLL and NTD^p24CA^ (PDB code: 2LF4). Ank^GAG^1D4 bound to helix 1 (**A**) Ank^GAG^1D4 bound to helix 7 (**B**). The atom style represents the residues on the NTD^p24CA^ (in ribbon style) interacting with Ank^GAG^1D4 (surface form). The colour of the atom style indicates the distance between the NTD^p24CA^ residues and Ank^GAG^1D4 in which pink, cyan, dark green, light green, yellow, and orange indicate distances at <5 Å, 5–10 Å, 10–20 Å, 20–30 Å, and 30–40 Å, >40 Å, respectively. The variable residue, S120 (40.46%), is outside the binding pocket residues in the case of Ank^GAG^1D4 bound to helices 1 and 7 of the NTD.

To analyse the NTD^p24CA^ amino acid distribution on the Ank^GAG^1D4 binding pocket residues, the docking complexes from those previously reported of Ank^GAG^1D4 bound to helix 1 of NTD^p24CA^ and helix 7 were utilised [25]. The sequence identity between the consensus amino acid and the p24CA structure in the docking complexes was 96.6%. Therefore, the Ank^GAG^1D4 binding pocket residues from those docking complexes could be applied in this study. In the case of the Ank^GAG^1D4 bound helix 1 of NTD^p24CA^, the Ank^GAG^1D4 binding pockets (18 residues) showed 17 (94.4%) highly conserved residues, as shown in by bullets in Figure 5. Ank^GAG^1D4 bound to helix 1 interacted with only one polymeric residue of NTD^p24CA^ in the binding interface in <5 Å at positions Q13, S16, P17, R18, T19, N21, A22, K25, V26, E29, K30, N33, P34, E35, V36, P38, M39, and L43. Although the rest of the amino acids had polymorphisms, they were not classified as variable amino acids, because the prevalence of the consensus amino acid (N33^91.6^) was not less than 50%. For the complex of Ank^GAG^1D4 bound to helix 7 of NTD^p24CA^, 26 out of the 31 residue positions (83.9%), as shown by the diamonds in Figure 5, in the Ank^GAG^1D4 binding pocket residues were highly conserved residues, whereas the rest of the amino acids were polymorphic residues (E79^90.1^, V83^54.2^, P123^71.8^, D128^91.6^, and L136^93.1^) that were not classified as variable residues. Ank^GAG^1D4 bound to helix 7 interacted with two polymeric residues of NTD^p24CA^ in the binding interface in <5 Å at positions N5, P38, M39, S41, A42, E45, E75, E76, E79, W80, R82, V83, T119, N121, P123, P125, D128, I129, K131, R132, W133, I135, M136, G137, L138, N139, K140, I141, V142, R143, and M144. Therefore, among 19 polymorphic amino acids of NTDp24CA, there were a few residues that were contained in the Ank^GAG^1D4 binding pocket that were not classified as variable residues (Figure 6).

## 4. Discussion

Developing an anti-HIV-1 drug is a remarkable therapeutic story in itself, with the challenge of increasing incidence of drug resistance [29,30]. Continuous efforts are required to demonstrate the efficacy of new HIV-1 inhibitors. Several inhibitors have been designed by targeting the conserved region, i.e., the p24CA domain of the Gag polyprotein [31,32,33,34,35]. The p24CA protein, which is a component of Gag, is required to form the structural core in mature viral particles [36,37]. The mature HIV-1 p24CA comprises two independently folded domains, NTD^p24CA^ and CTD^p24CA^, joined by a flexible linker. The interactions of NTD^p24CA^–NTD^p24CA^ and NTD^p24CA^–CTD^p24CA^ are essential for the assembly and stabilisation of the p24CA pentamer and hexamer rings. The p24CA hexamer [38] showed that helices 1–3 of each NTD^p24CA^ appear to form the NTD^p24CA^–NTD^p24CA^ interface, whereas helices 4 and 7 are involved in the NTD^p24CA^–CTD^p24CA^ interface (Figure 5). Thus, it was somewhat unsurprising that these regions contained high numbers of conserved residues or low polymorphism residues, regardless of the HIV-1 subtype, preserving its function [39,40].

Ank^GAG^1D4 has been reported to impair the HIV-1 assembly process, leading to a reduction in the production of viral progeny [12]. Besides the determination of the p24CA level on culture supernatant, the accumulation of the level of intracellular p24CA in SupT1/Ank^GAG^1D4, and SupT1/Ank^A3^2D3 cells on day 7 post-infection was demonstrated in Supplementary Figure S1. The intracellular p24CA level in SupT1/Ank^GAG^1D4 was lower than the SupT1/Ank^A3^2D3 control. The non-inhibited samples (DK071, and UU80) also obtained a high level of intracellular p24CA. The intracellular p24CA level corresponded with the p24CA level in the culture supernatant. This finding was concordant with our previous publication [12]. Regarding the recent report describing the host defense mechanism for HIV [41], we contemplate that the rate of HIV-1 Gag lysosomal degradation is higher than that of HIV-1 Gag biosynthesis in SupT1/Ank^GAG^1D4. The complex of p24CA and Ank^GAG^1D4 presumably promotes the BCA2 pathway. In addition, the maturation of virion harvested from various days after chimeric viruses challenging was subsequently examined. The viral load assay was performed (Supplementary Figure S2) along with the infectivity assay (Supplementary Figure S3). It showed that the %GFP in SupT1/Ank^GAG^1D4 was significantly lower than the SupT1/Ank^A3^2D3 control on day 9. These data substantiated that the function of Ank^GAG^1D4 is interfering the mature virion production. The Ank^GAG^1D4 expression that was observed in the present study could be considered a reasonable avenue for clinical application. Therefore, not only should third-generation self-inactivating vectors be designed and used, but also the most evolved features of the tetracycline (Tet)-inducible system should be combined to allow for the safe and efficient intracellular expression of the Ank^GAG^1D4 protein, which can be regulated by being switched on/off in further applications in HIV gene therapy [42,43].

In HIV-1 infection, the virus uses the host cellular machinery to produce viral components, including viral proteins and the viral genome, which consequently move to the plasma membrane, where they form a new particle and undergo budding. In addition, HIV-1 replicative fitness is normally controlled by the functions of the envelope (Env) protein [44,45,46]. Env was not involved in the viral fitness that was assessed in this study, because the chimeric viruses that were used carried a different Gag/PR-coding region from HIV-1-infected individuals instead of real HIV-1 viruses from the patients. Therefore, amino acid variations among Gag/PR proteins might affect the rate of viral replication and/or viral infectivity. In fact, the amino acid sequence analysis on NTD^p24CA^ in this study showed that although some sequences were identical (Table 1), the levels of p24CA that were detected from the samples in the SupT1/Ank^A3^2D3 non-binding control were still significantly different. For example, the Gag/PR chimeric viruses 015F and UU53 had 100% sequence identity; however, their p24CA levels were found to be 3.5 µg/mL and 937.5 µg/mL, respectively. As presented in the absence of Ank^GAG^1D4, the p24CA level detected in the culture supernatant fluids varied in the range of 2.1–1612.0 µg/mL. Viral fitness has influenced the p24CA level; however, the Ank^GAG^1D4 expression was still significantly reduced by p24CA production in all four clusters, (C1, C2, C3, and C4), as demonstrated in Figure 2 and Figure 3.

Microscopic observation of the infected cells revealed that the syncytia formation induced by virus infection correlated with the level of p24CA. For instance, the high p24CA level in Ank^A3^2D3 expressing SupT1 infected with DK001 (25.4 µg/mL) induced the formation of a higher number of syncytia than did the DK017 virus (5.5 µg/mL), as shown in Figure 4. Therefore, many of the formed syncytia, which could be found in the supT1/Ank^A3^2D3 non-binding control, could be associated with the function of the anti-HIV-1 effect by Ank^GAG^1D4.

The distribution of NTD^p24CA^ amino acids among the 131 chimeric NL4-3 viruses carrying Gag/PR-coding regions showed that 19 out of the 146 NTD^p24CA^ positions had polymorphism rates equal to or above 5% (blue superscripts in Figure 5). A few of these polymorphic residues are involved in the NTD^p24CA^–NTD^p24CA^ interface (positions 14, 15, and 20) and the NTD^p24CA^–CTD^p24CA^ interface (position 71) to form the p24CA hexamer, of which only position 71 (E^89.3^ and D^10.7^) made a hydrogen bond to CTD^p24CA^. However, polymorphisms at this position may not affect stabilisation with CTD^p24CA^, because glutamic acid and aspartic acid belong to the same group of amino acids. In clusters C2 and C3, presenting a clear high fold change in the p24CA (2D3:1D4) ratio, the alternative amino acids of positions 14 (P, S, A), 15 (L, V, I, F), 20 (M, L), and 71 (E, D) may not interfere with the NTD^p24CA^–NTD^p24CA^ interface and the NTD^p24CA^–CTD^p24CA^ interface. Thus, we suggest that the polymorphic residues do not have an influence on the formation of the p24CA hexamer. For the docking structure of NTD^p24CA^–Ank^GAG^1D4, the Ank^GAG^1D4 binding pocket residues with polymorphisms (Ank^GAG^1D4 bound to helix 1, residue N33; otherwise, residues E79, V83, P123, D128, and M136) are also hypothesised not to affect the interaction with Ank^GAG^1D4, as seen in the significantly different p24CA level between SupT1/Ank^GAG^1D4 and SupT1/Ank^A3^2D3 (Figure 3). Thus, the natural polymorphisms of the NTD^p24CA^ residues that are contained inside and outside the Ank^GAG^1D4 binding pocket residues (Figure 6) did not impair Ank^GAG^1D4’s function. This indicated that Ank^GAG^1D4 could be used against a broad spectrum of HIV-1 subtypes in terms of sensitivity to natural polymorphisms.

Relying on peptide scanning and mutagenesis, combined with ELISA, the target regions of Ank^GAG^1D4 were identified as helices 1 and/or 7 of NTD^p24CA^, and especially the key binding residues R18, R132, and R143 [25]. Interestingly, the Ank^GAG^1D4 binding pocket, which is important for Gag polymerisation, contains low levels of polymorphism in several HIV-1 strains. Nonetheless, the negative interference in the HIV-1 particle assembly by Ank^GAG^1D4 has been tested only against the HIV-1_NL4-3_ laboratory strain (91.1% sequence identity to consensus sequence of the 131 individuals used here, data not shown). Therefore, in this study, the observation of Ank^GAG^1D4 activity in HIV-1 assembly on several circulating strains revealed the broad anti-HIV-1 effect of Ank^GAG^1D4.

By structural analysis, the Ank^GAG^1D4 binding pocket was found to comprise 18 and 31 NTD^p24CA^ residues in the docking complex of Ank^GAG^1D4 bound to helices 1 and 7, respectively [25]. In the case of Ank^GAG^1D4 bound to helix 1 of NTD^p24CA^, most of the binding pocket residues were highly conserved residues; in particular, conserved R18 was an important amino acid for the NTD^p24CA^–NTD^p24CA^ interface [38,47] and the NTD^p24CA^–Ank^GAG^1D4 interface [25]. Similarly, when binding to helix 7, 83.9% of the residues in the binding pocket were highly conserved. Although residues R132 and R143 did not directly interact with CTD^p24CA^, the huge size of Ank^GAG^1D4 hindered the formation of the helix 7–CTD^p24CA^ interface and the movement of the flexible linker that allows the rearrangement of CTD^p24CA^ to interact with NTD^p24CA^ [37]. Thus, the unsuccessful polymerisation of Gag was the result of the interference with the NTD^p24CA^–NTD^p24CA^ and the NTD^p24CA^–CTD^p24CA^ interfaces by Ank^GAG^1D4.

The sequence variation in NTD^p24CA^ was low, resulting in 96.1% sequence identity between viral sequences from the 131 individuals. However, among the 131 sequences, there were 115 unique sequences in which 13% polymorphism was observed among 146 NTD^p24CA^ positions. Of the amino acids having polymorphism, only one residue was a variable residue (S120). This result is similar to the NTD^p24CA^ distribution of natural polymorphism of the HIV-1 group M (subtypes A1, B, C, D, F1, G, and CRF01_AE, CRF02_AG), which were reported to have 17.8% polymorphisms and three variable amino acids (A14, V27, and N120) [39]. More importantly, there was no variable residue present among the Ank^GAG^1D4 binding pocket residues, especially in the key interacting residues (R18, R132, and R143). Interestingly, these conserved residues were also observed in two non-inhibited viruses (DK071 and UU80). We also found that the NTD^p24CA^ of DK071 and UU80 are closely similar to the subtype B NL4-3 viruses by 90.4% and 91.7%, respectively, whereas the lowest sequence identity 66.4% of CRF01_AE, UU01, was also inhibited by Ank^GAG^1D4. Therefore, other factors are prone to influence the observed phenomenon for the non-inhibiting of DK071 and UU80 viruses. The supportive information of Ank^GAG^1D4 reacting with highly conserved regions (R18, R132, and R143) of NTD^p24CA^ was provided in Supplementary Figure S4. The results obtained from the NTD^p24CA^ sequences in this study implied that Ank^GAG^1D4 is suitable for use in a wide spectrum of HIV-1 strains.

## 5. Conclusions

The efficient inhibitory effect of Ank^GAG^1D4-expressing cells on Gag/PR chimeric HIV-1 that was observed in this study strongly supports the application of the ankyrin repeat protein as a broad-target, alternative, therapeutic molecule for HIV-1 gene therapy.

## Figures and Tables

**Table 1:** Sequence Identity of Five Identical Sequences in 16 Individuals.

Type	AA Position	Sequence Identity (%)						Individual	p24CA Level (µg/mL)	
		ccAA	IS#1	IS#2	IS#3	IS#4	IS#5		Ank^A3^2D3	Ank^GAG^1D4
IS#1	-	100	100	99.3	99.3	97.9	97.3	015F	3.5	2.3
								UU53	937.5	479.3
IS#2	L15V	99.3	99.3	100	98.6	98.6	97.9	UU51	92.4	14.8
								UU23	511.1	440.1
IS#3	S120N	99.3	99.3	98.6	100	97.2	97.3	004F	58.6	4.9
								025M	2.3	2.1
								041F	6.2	2.2
								044M	18.7	2.9
								046F	563.9	487.4
								049M	3.3	2.4
								DK056	148.8	8.4
								UU06	6.4	3.1
IS#4	L15V, E79D, V83L	97.9	97.9	98.6	97.2	100	96.6	DK029	8.1	2.4
								DK063	64	11.1
IS#5	L15V, V83L, G91N, P123S	97.3	96.6	97.9	97.9	97.3	100	DK001	25.4	2.3
								DK002	4.1	2.2

Note: ccAA is defined as the corresponding consensus amino acid from 131 p24CA sequences.

**Table 2:** NTD^p24CA^ Amino Acid Distribution of Key Residues Interacting with Ank^GAG^1D4 in 11 Individuals.

NTD^p24CA^ #	18	132	143
**Residue**	**R**	**R**	**R**
012M	T	-	-
DK064	T	-	-
DK007	T	-	-
DK050	T	-	-
DK051	K	-	-
UU08	K	-	-
UU01	S	-	-
040F	I	I	-
DK032	-	K	K
UU27	-	-	G
UU049	-	-	K

Note: NTD^p24CA^**#** is the amino acid index.

## Supplementary Materials

The supplementary materials are available at http://www.mdpi.com/1999-4915/10/11/625/s1. Figure S1: The intracellular levels of p24CA in SupT1/Ank^GAG^1D4, and SupT1/Ank^A3^2D3 cells on day 7 post-infection. Figure S2: The viral load analysis from culture supernatants of SupT1/Ank^GAG^1D4 and SupT1/Ank^A3^2D3 infected with individual viruses. Figure S3: The infectivity of released virion. Figure S4: The NTD^p24CA^ residue comparison of the chimeric viruses DK071, UU80, and UU01.
