# Supplementary material for: Broad-Spectrum Antiviral Activity of an Ankyrin Repeat Protein on Viral Assembly against Chimeric NL4-3 Viruses Carrying Gag/PR Derived from Circulating Strains among Northern Thai Patients
Source: Viruses. 2018 Nov 13;10(11):625. doi: 10.3390/v10110625 (PMC6265948; doi:10.3390/v10110625)
Supplement: Supplementary file 1 [file viruses-10-00625-s001.pdf]

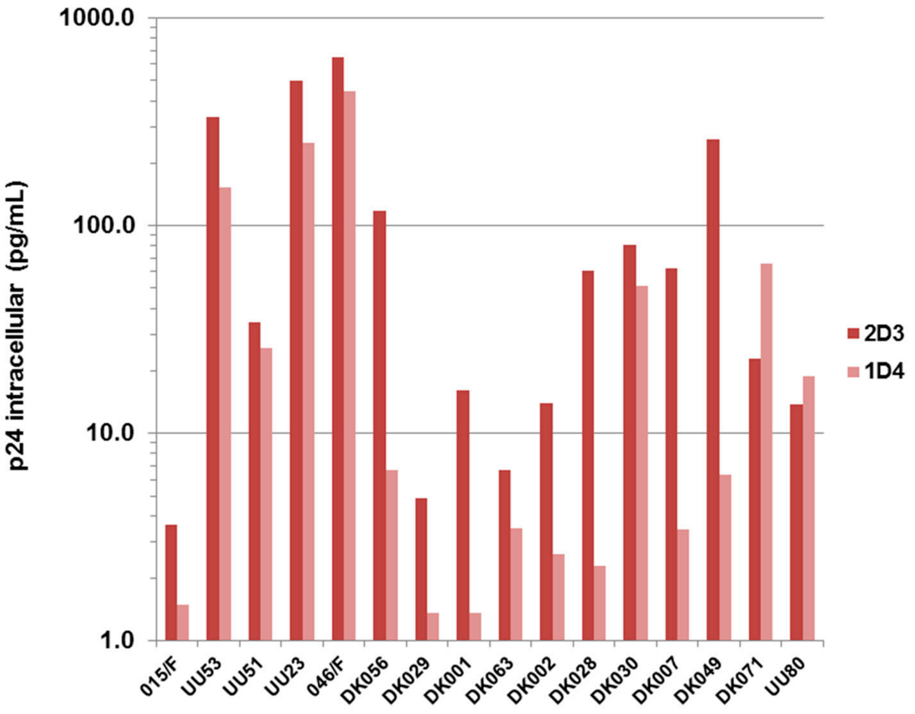

Figure S1. The intracellular levels of p24CA in SupT1/Ank^{GAG}1D4, and SupT1/Ank^{A3}2D3 cells on day 7 post-infection. The SupT1/Ank^{GAG}1D4 and SupT1/Ank^{A3}2D3 cells were collected on day 7 post-infection for this purpose. The representative 14 samples in each cluster (C1, C2, C3, and C4) and two samples (DK071 and UU80), for which Ank^{GAG}1D4 did not show the inhibition effect, were selected. Cellular proteins were extracted by RIPA buffer. The total protein lysates were normalised to 10 μg/mL to determine the intracellular levels of p24CA using Genscreen ULTRA HIV Ag-Ab Assay.

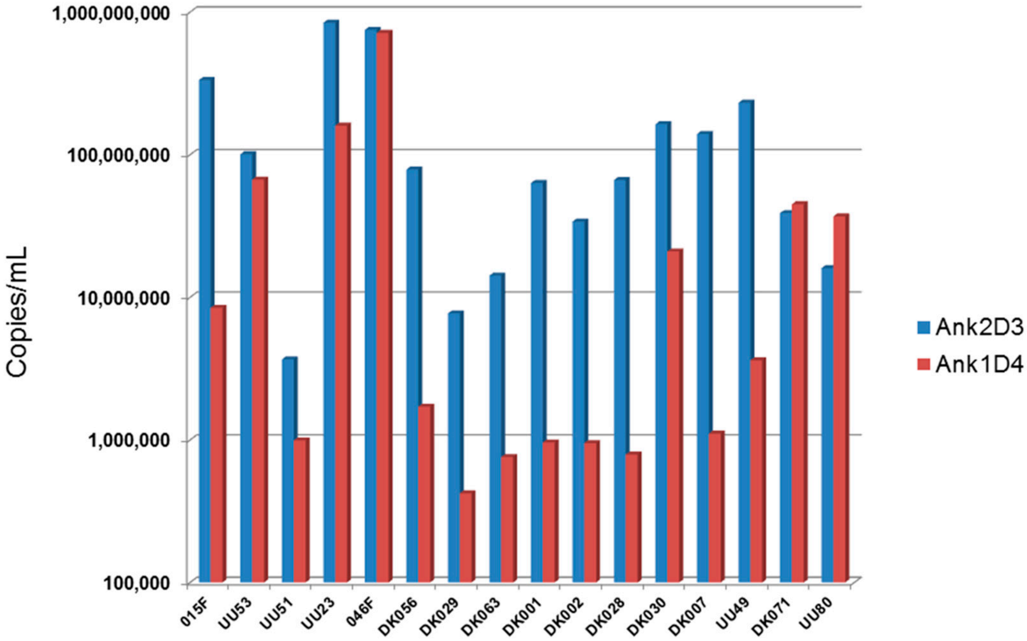

Figure S2. The viral load analysis from culture supernatants of SupT1/Ank[GAG]1D4 and SupT1/Ank[A3]2D3 infected with individual viruses. The representative 14 samples in each cluster (C1, C2, C3, and C4) and two samples (DK071 and UU80), which Ank[GAG]1D4 did not show the inhibition effect, were selected. The level of viral genomic RNA collected on day 7 post-infection was determined using COBAS AmpliPrep/COBAS TaqMan HIV-1 test V2.0.

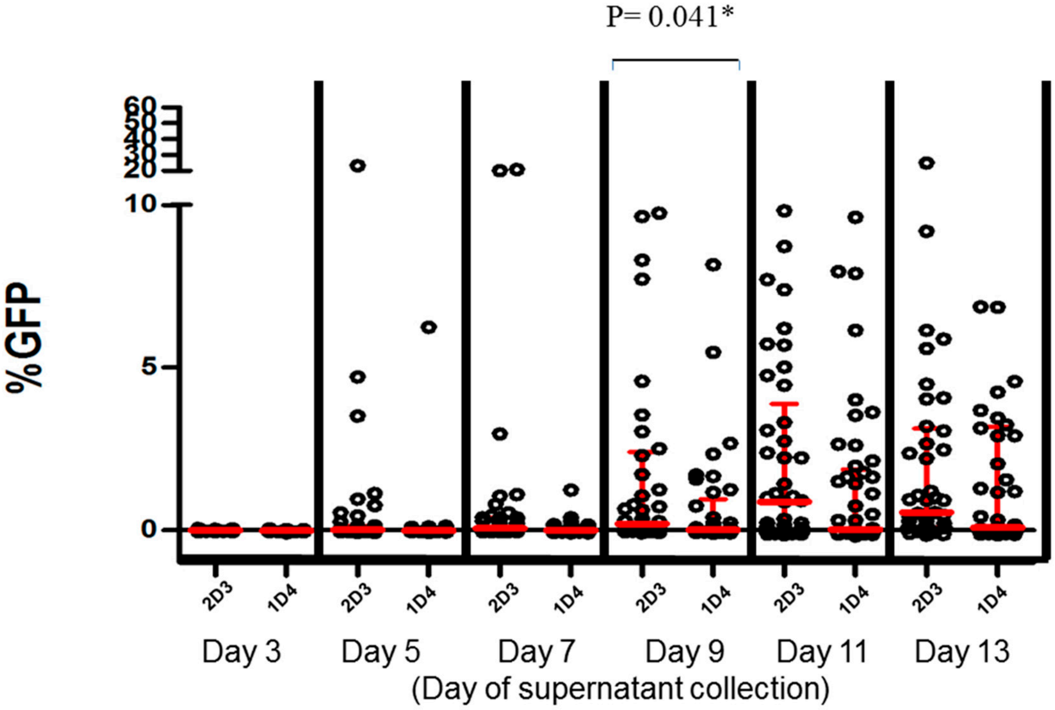

Figure S3. The infectivity of released virion. The tat-driven GFP reporter Jurkat T-cells (kindly gifted by Dr. Toshiyuki Miura) were challenged with viral supernatants harvested from various days post

chimeric HIV-1 infection. After three days of infection, these cells were harvested for flow cytometry. The percentage of green fluorescent protein (GFP)-positive cell was statistically analysed by Mann–Whitney U test/ Wilcoxon W test.

A)

	Amino acid position 18	Amino acid position 132	Amino acid position 144
Consensus	R	R	R
DK071	R	R	R
UU80	R	R	R

B)

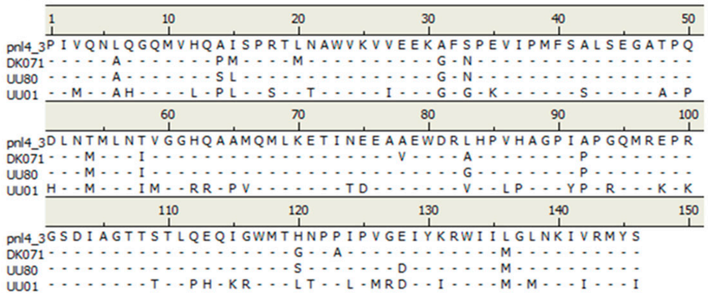

Sample No.	Sequence identity to pNL4-3 (%)
DK071	90.4
UU80	91.7
UU01	66.4

Figure S4. The NTD^{p24CA} residue comparison of the chimeric viruses DK071, UU80, and UU01. (**A**) The Arginine (R) residues 18, 132, and 143 of DK071 and UU80 were depicted. (**B**) The NTD^{p24CA} sequence alignment of DK071, UU80, and UU01 against subtype B NL4-3 virus. The percentage of the sequence identity was shown.
